# Coronaviruses Among Swine Workers in Northern Vietnam

**DOI:** 10.1111/irv.13293

**Published:** 2024-04-15

**Authors:** Laura A. Pulscher, Duy Tung Dao, Samantha G. Cody, Victor DelPrincipe, Gregory C. Gray

**Affiliations:** ^1^ Division of Infectious Diseases, Department of Medicine University of Texas Medical Branch Galveston Texas USA; ^2^ Virology Department National Institute of Veterinary Research Hanoi Vietnam; ^3^ Institute for Translational Science University of Texas Medical Branch Galveston Texas USA; ^4^ Department of Microbiology and Immunology University of Texas Medical Branch Galveston Texas USA; ^5^ Institute for Human Infections and Immunity University of Texas Medical Branch Galveston Texas USA; ^6^ Department of Global Health and Emerging Diseases, School of Public and Population Health University of Texas Medical Branch Galveston Texas USA

Dear Editor,

The SARS‐CoV‐2 pandemic has highlighted the significant clinical relevance of coronaviruses, and the emergence of these pathogens has been associated with zoonotic transmission from animal reservoirs [1]. This has prompted enhanced surveillance for novel viral isolates especially in critical spillover settings, particularly at the human–animal interface. Coronaviruses circulate in many species of mammals, including pigs, and while not well understood, recent evidence suggests that swine coronaviruses (SCoVs) may be spilling over into the human population. For instance, a recent study reported spillovers of porcine deltacoronavirus (PDCoV) into children in Haiti with undifferentiated febrile illness, suggesting PDCoV possesses the ability to infect humans [2]. Another SCoV that has drawn much attention for its potential zoonotic nature is swine acute diarrhea syndrome coronavirus (SADS‐CoV). While not reported in humans, SADS‐CoV can infect various cell lines, including human cell lines, and has also been reported to infect various experimental animals [3]. Together, these studies suggest that SCoVs have potential to become an emerging threat for human health. To further understand the prevalence and potential spillover of novel CoVs, we conducted a study of CoVs among swine workers in northern Vietnam.

From June 2019 to May 2020, as part of an influenza A virus surveillance study, we collected nasal washes from 401 swine workers on five swine farms in northern Vietnam as previously described [4, 5]. Briefly, study team members performed monthly sampling visits to each farm and, after consent, collected nasal washes from swine workers by injecting 5 mL of sterile water into one nostril and collecting the expressed fluid. Samples were then transported to National Institute of Veterinary Research (NIVR) where they were aliquoted and stored at −80°C until shipped to the University of Texas Medical Branch (UTMB) for study. Human subject research was approved by ethics committees at Duke University, Duke‐NUS Medical School, and the Hanoi University of Public Health, Hanoi, Vietnam. Institutional Animal Care and Use Committee approval was granted by Duke University.

RNA extraction was conducted using a QIAamp Viral RNA mini kit (Qiagen, Valencia, CA) either by hand or on a QIACube Connect (Qiagen, Valencia, CA) per the manufacturer's instructions. Extracted samples were screened for coronaviruses using a gel‐based conventional semi‐nested RT‐PCR targeting the RNA‐dependent RNA polymerase (RdRp) genome [6] using Superscript III Platinum One‐Step RT‐PCR System with Platinum Taq DNA Polymerase (Thermo Fisher Scientific, Inc., Waltham, MA). Samples positive for coronaviruses were sent for amplicon sequencing and assessed for sequence similarity to other coronaviruses using the National Center for Biotechnology–Basic Local Alignment Search Tool (NCBI Blast). Phylogenetic analysis was performed using Geneious Prime software V 2023.2.1 (Dotmatics, Boston, Massachusetts).

Three (0.75%) swine workers had molecular evidence of CoVs. Alignment of these sequences in NCBI blast showed close identity to human coronavirus 229E strain 229E/Russia/SPE‐RII‐25806S/2021 (Figure 1). No other samples were positive for CoVs. There is little information on seasonal CoV strains, particularly 229E, among Vietnamese populations. However, the low prevalence (~1% prevalence) of 229E in this study is within the prevalence range reported for other seasonal CoVs, OC43 and NL63, in the limited studies conducted among adult Vietnamese populations [7, 8]. Despite us studying swine workers prospectively over the course of a year, we did not detect any spillover of SCoVs. Although we did not test swine samples in this study, it is possible that SCoV prevalence among the swine herds on these farms is low. Interestingly, we had low detections of other respiratory diseases including Influenza A virus [5] and Influenza D virus [4] among these swine herds and their workers previously despite seeing a high prevalence of influenza A viruses among poultry in the same areas [5]. This suggests that there may be a low prevalence of influenza and CoVs among the swine workers, and possibly their swine, potentially due to increased biosecurity protocols among these farms [4]. Regardless, future studies should be conducting with a larger cohort of livestock workers and their animals to further understand the threat of novel CoVs spilling over into humans.

**FIGURE 1:**
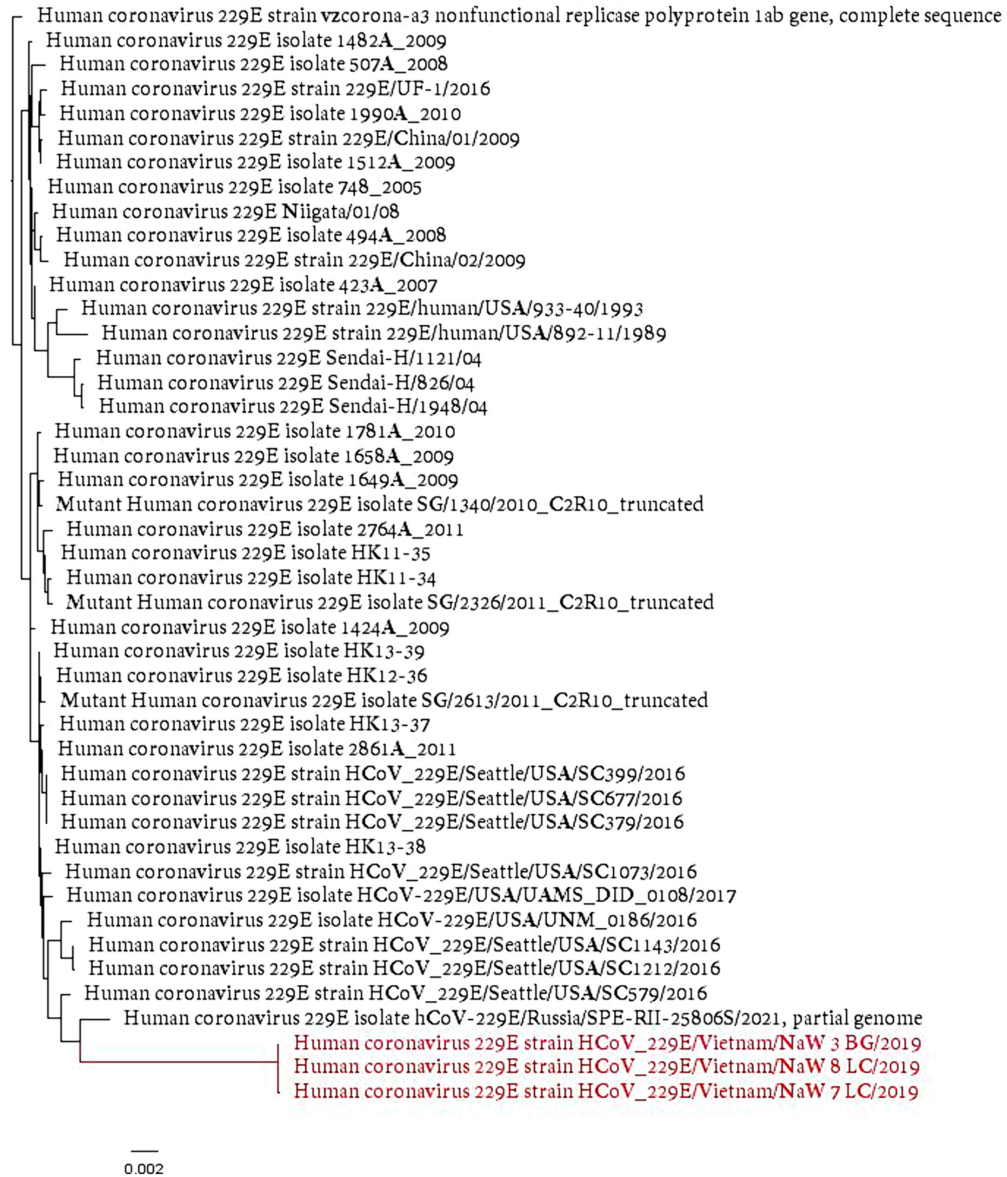
A neighbor‐joining phylogenetic tree of a partial RNA‐dependent RNA polymerase (RdRp) sequence of the human coronavirus 229E subtypes (GenBank accession numbers PP496883–PP496885) identified in three human nasal washes. The partial RdRp sequences identified in this study are indicated in red and representative human coronavirus 229E sequences from GenBank are indicated in black.

## Author Contributions

**Laura A. Pulscher:** Writing – original draft; Methodology; Investigation; Validation; Formal analysis; Data curation. **Duy Tung Dao:** Investigation; Methodology; Data curation; Validation; Writing – review and editing. **Samantha G. Cody:** Investigation; Methodology. **Victor DelPrincipe:** Methodology; Investigation. **Gregory C. Gray:** Conceptualization; Methodology; Supervision; Writing – review and editing; Funding acquisition.

## Conflicts of Interest

The authors declare no conflicts of interest.

### Peer Review

The peer review history for this article is available at https://www.webofscience.com/api/gateway/wos/peer‐review/10.1111/irv.13293.

## Data Availability

The data that support the findings of this study are available from the corresponding author upon reasonable request.
